# Supplementary material for: Insertion of a Clinical Pathway Pop-Up Window into a Computer-Based Prescription System: A Method to Promote Antibiotic Stewardship in Upper Respiratory Tract Infection
Source: Antibiotics (Basel). 2021 Dec 2;10(12):1479. doi: 10.3390/antibiotics10121479 (PMC8698948; doi:10.3390/antibiotics10121479)
Supplement: Supplementary file 1 [file antibiotics-10-01479-s001.zip › antibiotics-1420744-supplementary.pdf]

Supplemental Data

Table S1: Factors associated with rational antibiotic prescription

Factor	cOR (95%CI)	p-value
Sex (male)	1.19 (0.82-1.74)	0.365
DM	1.59 (0.53-4.81)	0.408
HT	0.49 (0.22-1.08)	0.078
Airway disease	0.83 (0.25-2.72)	0.757
Steroid use	1.11 (0.22-5.66)	0.898
Fever	1.22 (0.84-1.78)	0.303
Cough*	0.45 (0.26-0.77)	0.004
CLN tenderness	1.92 (0.65-5.65)	0.236
Exudate on tonsil(s)	0.79 (0.43-1.43)	0.430
Facial tenderness	0.76 (0.35-1.66)	0.486
Purulent nasal discharge	0.51 (0.21-1.24)	0.138
History of recurrent URI within 6 months	0.71(0.46-1.10)	0.129
Age ≥40	1.08 (0.73-1.60)	0.712
Physician's age ≥30*	0.47 (0.32-0.70)	<0.001
Setting (reference = general OPD)		
• Student PCU	0.97 (0.49-1.94)	0.939
• ED	0.84 (0.41-1.73)	0.631
• Premium clinic*	0.41 (0.18-0.89)	0.025
• ENT OPD	0.88 (0.36-2.14)	0.878
• Community PCU*	4.02 (1.06-15.20)	0.041
• Medicine OPD	2.22 (0.25-19.76)	0.475
Type of physician (reference = GP)		
• Resident	0.83 (0.54-1.29)	0.411
• Specialization other than internal medicine$, *	0.33 (0.20-0.53)	<0.001
• Internist	0.53 (0.19-1.46)	0.219
• Family doctor*	0.03 (0.00-0.27)	0.001
• ID physician**	NA	NA
**Clinical pathway use***	**2.36 (1.63-3.43)**	**<0.001**

CLN: cervical lymph node, CI: confidence interval, cOR: crude odds ratio, DM: diabetes mellitus, ED: emergency department, ENT: ear nose and throat, GP: general practitioner, HT: hypertension, ID: infectious disease, NA: not available, OPD: outpatient department, PCU: primary care unit, URI: upper respiratory infection

*Factor with statistical significance

**Number too low to calculate

$ "Specialization other than internal medicine" included *otolaryngologist, radiologist, rehabilitation physician, and psychiatrist*

Table S2: Factors associated with rational antibiotics prescription (multivariate analysis) when adjusted for physician's age, setting, physician type, and clinical pathway usage

Factors	aOR (95%CI)	p-value
Physician's age ≥30	1.56 (0.52-4.72)	0.429
**Clinical pathway usage***	**2.44 (1.61-3.70)**	**<0.001**
Type of physician (Reference = GP)		
• Resident	0.74 (0.45-1.23)	0.250

• Specialization other than internal medicine[$]<br>• Family doctor*	0.25 (0.07-0.90)<br>0.01 (0.00-0.12)	0.034<br><0.001
Location (Reference = general OPD)<br>• Premium clinic<br>• Community PCU*	 0.92 (0.30-2.84)<br>10.50 (1.96-56.27)	 0.882<br>0.006

aOR: adjusted odds ratio, CI: confidence interval, GP: general practitioner, OPD: outpatient department, PCU: primary care unit

*Factor with statistical significance

[$] Specialization other than internal medicine included *otolaryngologist, radiologist, rehabilitation physician, and psychiatrist*

Figure S1: Clinical pathway pop-up windows (Above: a pop-up with checkboxes for acute nasopharyngitis and acute sinusitis, below: a pop-up with checkboxes for acute tonsilitis and acute pharyngitis)

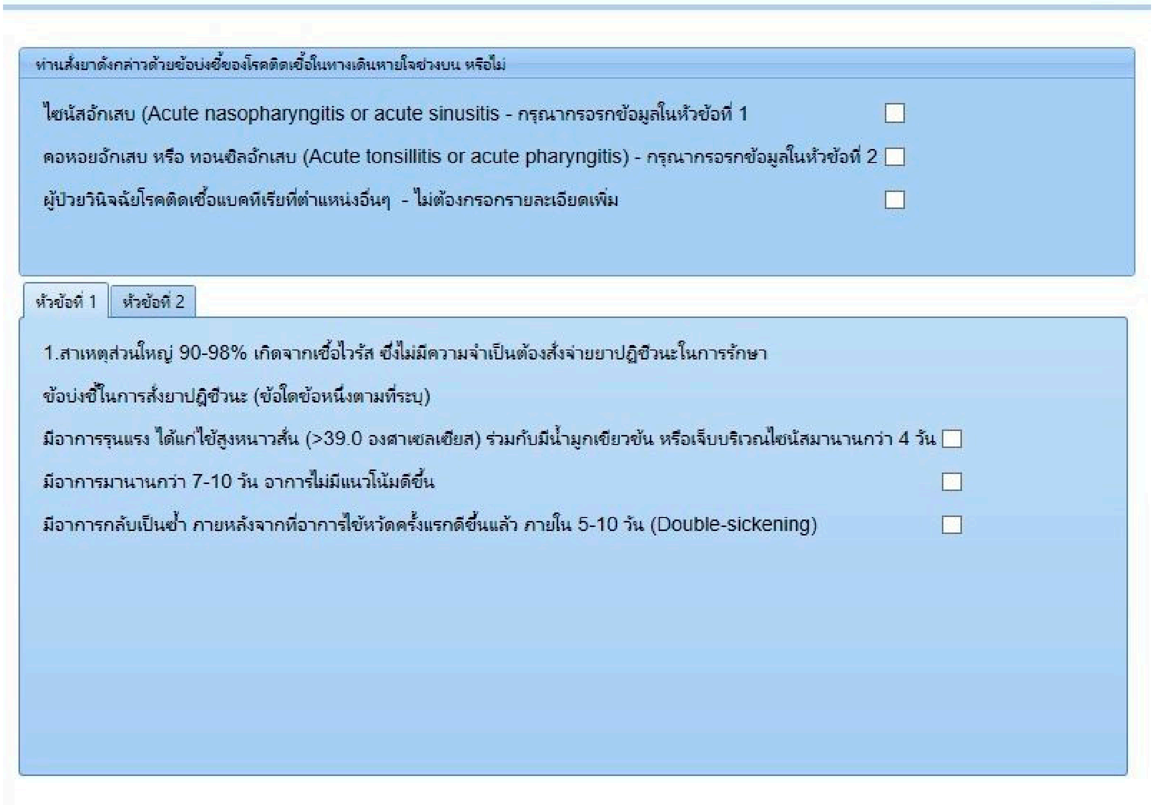

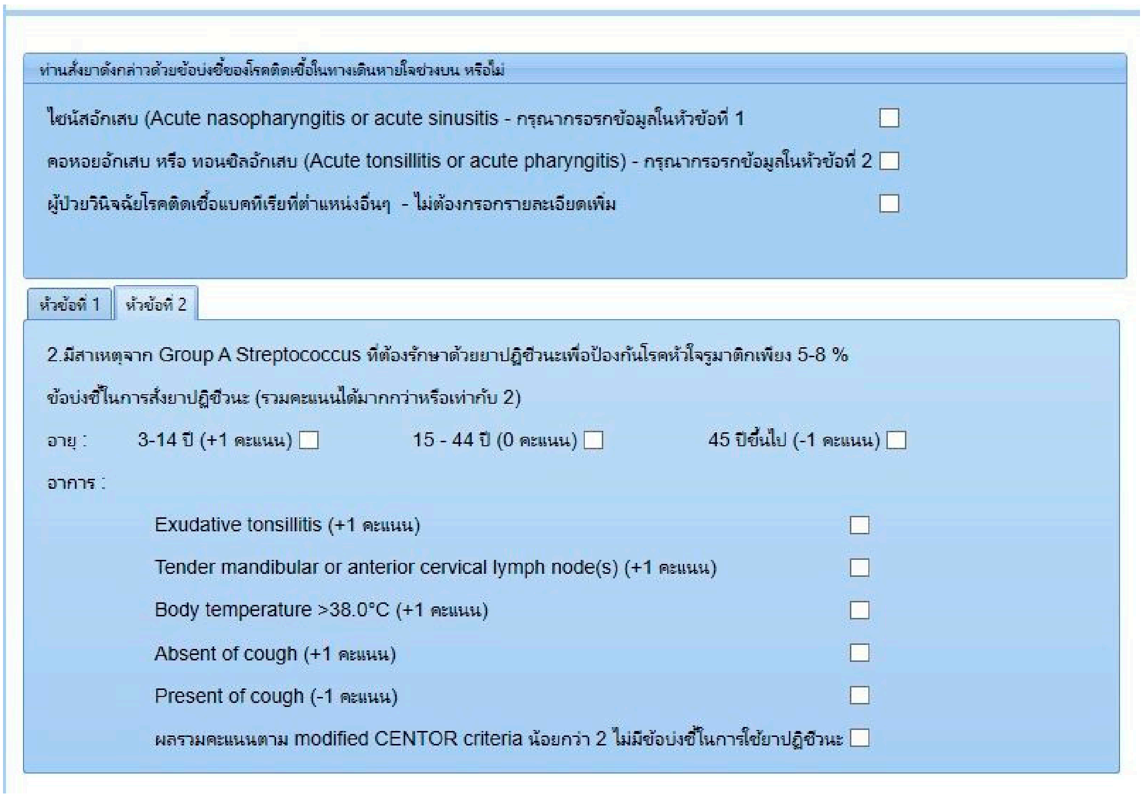

Figure S2: English translation of clinical pathway pop-up windows (Above: a pop-up with checkboxes for acute nasopharyngitis and acute sinusitis, below: a pop-up with checkboxes for acute tonsilitis and acute pharyngitis)

Are you prescribing this antibiotic for upper respiratory tract infection?

☐ Yes, for acute nasopharyngitis or acute sinusitis (Fill in section 1)
☐ Yes, for acute tonsillitis or acute pharyngitis (Fill in section 2)
☐ No, this antibiotic for other bacterial infectious diseases

Section 1: 90 to 98% of rhinosinusitis cases are caused by viral infection, for which antibiotic treatment is not necessary.

Indication for antibiotic (choose one of the following)

€ Symptoms or signs lasting for at least 7-10 days without clinical improvement

€ Severe symptoms (defined as fever ≥39°C and purulent nasal discharge or facial pain) lasting for at least 4 consecutive days at the beginning of illness

€Worsening of symptoms or signs following a typical viral URI that lasted 5–10 days and

Are you prescribing this antibiotic for upper respiratory tract infection?

- Yes, for acute nasopharyngitis or acute sinusitis (Fill in section 1)
- Yes, for acute tonsillitis or acute pharyngitis (Fill in section 2)
- No, this antibiotic for other bacterial infectious diseases

Section 2: Only 5-8% of acute pharyngitis cases are caused by group A streptococci and require antibiotic treatment to prevent rheumatic heart disease.

Antibiotic is indicated when total score ≥2

Age: € 3-14 (+1 point) € 15-44 (0 point) €≥45 (-1 point)

€Exudative tonsilitis (+1 point)

€Tender mandibular or anterior cervical lymph node(s) (+1 point)

€Body temperature >38.0°C (+1 point)

€Absence of cough (+1 point)

€ Presence of cough (-1 point)
